# Evaluation of external RNA controls for the standardisation of gene expression biomarker measurements

**DOI:** 10.1186/1471-2164-11-662

**Published:** 2010-11-24

**Authors:** Alison S Devonshire, Ramnath Elaswarapu, Carole A Foy

**Affiliations:** 1 LGC Limited, Queens Road, Teddington, Middlesex, TW11 0LY, UK

## Abstract

**Background:**

Gene expression profiling is an important approach for detecting diagnostic and prognostic biomarkers, and predicting drug safety. The development of a wide range of technologies and platforms for measuring mRNA expression makes the evaluation and standardization of transcriptomic data problematic due to differences in protocols, data processing and analysis methods. Thus, universal RNA standards, such as those developed by the External RNA Controls Consortium (ERCC), are proposed to aid validation of research findings from diverse platforms such as microarrays and RT-qPCR, and play a role in quality control (QC) processes as transcriptomic profiling becomes more commonplace in the clinical setting.

**Results:**

Panels of ERCC RNA standards were constructed in order to test the utility of these reference materials (RMs) for performance characterization of two selected gene expression platforms, and for discrimination of biomarker profiles between groups. The linear range, limits of detection and reproducibility of microarray and RT-qPCR measurements were evaluated using panels of RNA standards. Transcripts of low abundance (≤ 10 copies/ng total RNA) showed more than double the technical variability compared to higher copy number transcripts on both platforms. Microarray profiling of two simulated 'normal' and 'disease' panels, each consisting of eight different RNA standards, yielded robust discrimination between the panels and between standards with varying fold change ratios, showing no systematic effects due to different labelling and hybridization runs. Also, comparison of microarray and RT-qPCR data for fold changes showed agreement for the two platforms.

**Conclusions:**

ERCC RNA standards provide a generic means of evaluating different aspects of platform performance, and can provide information on the technical variation associated with quantification of biomarkers expressed at different levels of physiological abundance. Distinct panels of standards serve as an ideal quality control tool kit for determining the accuracy of fold change cut-off threshold and the impact of experimentally-derived noise on the discrimination of normal and disease profiles.

## Background

Transcriptomic approaches such as gene expression microarrays are being used routinely in diverse fields of research, such as toxicology and cancer biology, in order to characterize biological processes and find biomarkers indicative of pathological states and processes [1-3]. Compared to traditional clinical outcome measurements where a single biochemical measurement or histopathological score is interpreted, gene expression signatures resulting from microarray experiments generate a molecular fingerprint consisting of multiple biomarkers which cannot otherwise be interpreted in isolation. This approach has been applied successfully in the area of breast cancer prognosis, where the first *in vitro *diagnostic multi-variate index assay (IVDMIA) using gene expression measurements, MammaPrint (a microarray-based expression profile of 70 genes [5]), was approved for use by the FDA in 2007 [4], while OncotypeDx, a reverse-transcription quantitative PCR (RT-qPCR) -based assay profiles 21 genes in proliferation and estrogen receptor-related pathways [6].

In order to expedite the approval of new recognized biomarkers in other areas of diagnostics and in drug discovery, regulatory bodies such as the FDA and EU commission have highlighted the need to standardize results from 'omics' platforms using reference materials (RMs) in order to aid regulatory decisions (FDA Critical Path Opportunities List) [7]. It has been reported that better standardization during initial processes of biomarker identification would also improve the interpretation of meta-analyses where studies have been performed using different experimental protocols, platforms and designs [8,9]. Upon approval of multigene biomarker tests for clinical applications, reference materials would play an integral part in ongoing quality control (QC) procedures and proficiency testing (PT) in clinical laboratories [10].

At the moment standardized reference materials for assessing results from different gene expression platforms are lacking [9]. In the field of gene expression microarrays, array manufacturers usually supply controls which can provide information on platform performance and ascertain the quality of sample labelling and hybridization procedures. However, platform-specific do not allow direct comparison of gene expression data from different platforms, leading to calls for universal microarray reference materials [11,12]. Gene expression studies by RT-qPCR techniques often use reference gene levels as a means for assessing sample processing and normalising for the mRNA content of a sample [13]. However, reference gene expression is often influenced by the experimental conditions under investigation, requiring thorough validation of their stability [14,15]. In contrast, *in vitro *produced RNA spike-ins are independent of the biological process and can act as controls for both the RT and qPCR steps [13,16]. Artificial spike-ins are also useful for assessing the presence of inhibitors from the sample in the RT-qPCR reactions [17]. However, like microarray spike-ins, cross-platform RNA standards are not widely available for RT-qPCR applications.

Efforts to address these issues are in progress through the ERCC initiative for developing a large set of publicly available RNA standards [18]. A panel of 96 different standards developed through the ERCC project consists of artificial sequences or sequences from bacterial and other genomes which lack homology to human sequences [19]. These RNA standards have been produced by *in vitro *transcription (IVT) and contain synthetic 3' polyA sequences, which enable them to be processed in the same way as mRNA transcripts, using oligo(dT)-based priming strategies which are commonly used in microarray sample labelling and RT-qPCR protocols.

In this study we tested panels of selected RNA standards with potential application for biomarker validation for the two most commonly used technologies for gene expression quantification-DNA microarrays and RT-qPCR. The study focussed mainly on the detection properties of the standards using Agilent one-colour oligonucleotide microarrays and Taqman^® ^real-time PCR methods. The standards were used to investigate some of the performance characteristics on two representative platforms, namely linear dynamic range, limit of detection (LOD) and technical reproducibility. We also developed two separate panels of these standards designed to mimic 'normal' and 'disease' states, where some biomarkers are differentially expressed whilst others remain unchanged in their expression. Finally, we have demonstrated the use of such panels informed decision-making regarding fold change cut-off thresholds and assessment of the impact of technical factors on the discrimination between control and treatment groups.

## Results

### Characterisation of gene expression platforms using RNA standards

Our initial aim was to demonstrate the use of universal RNA standards to characterize different methods for gene expression quantification and provide technical information which can be applied to mRNA biomarkers of differing levels of abundance. In order to closely mimic biological scenarios [20], RNA standards were spiked into human total RNA prior to setting up microarray labelling or reverse transcription reactions. Eight different standards, ranging in length from 481 to 1324 bases, and varying composition of GC bases were selected to provide balanced differences in reaction efficiencies due to transcript sizes and secondary structure considerations (Additional File 1).

The detection of eight RNA standards was investigated using common microarray and RT-qPCR approaches- Agilent one-colour 4 × 44 K microarrays and Taqman^®^-based real-time PCR using ABI 7900HT real-time PCR system, incorporating in- house designed oligonucleotide probes and assays respectively (for sequences, see Additional File 1). In the first of two experiments, copy number for RNA standards was varied across seven orders of magnitude between 1 and 10^6 ^copies per ng total RNA, in a background of Universal Human Reference RNA (Stratagene) (Table 1). Copy numbers were chosen to extend over the natural physiological levels of transcript abundance, approximating to a range of between 0.01 and 10^4 ^copies of an individual mRNA transcript per mammalian cell with a total RNA content of 26 pg [21]. A negative control with zero copies of each standard was also included in each sample in order to measure background signals and check the specificity of the assays.. The composition of each sample was balanced to contain the same total number of transcript copies (Table 1) and the addition of spike-in materials did not increase the mRNA content of the sample by more than 4% (assuming mRNA as 2% of total RNA [21]).

**Table 1:** Composition of pooled samples (numbers denote copies/ng total RNA)

	**Sample pool no**.							
**RNA standard**	**1**	**2**	**3**	**4**	**5**	**6**	**7**	**8**

**13**	1	10	10^2^	10^3^	10^4^	10^5^	10^6^	0

**42**	0	1	10	10^2^	10^3^	10^4^	10^5^	10^6^

**81**	10^6^	0	1	10	10^2^	10^3^	10^4^	10^5^

**84**	10^5^	10^6^	0	1	10	10^2^	10^3^	10^4^

**95**	10^4^	10^5^	10^6^	0	1	10	10^2^	10^3^

**99**	10^3^	10^4^	10^5^	10^6^	0	1	10	10^2^

**113**	10^2^	10^3^	10^4^	10^5^	10^6^	0	1	10

**171**	10	10^2^	10^3^	10^4^	10^5^	10^6^	0	1

Eight different RNA standards were mixed in a background of Stratagene Universal Reference RNA across a range of copy numbers between 0 and 10^6 ^copies/ng total RNA.

**Figure 1:**
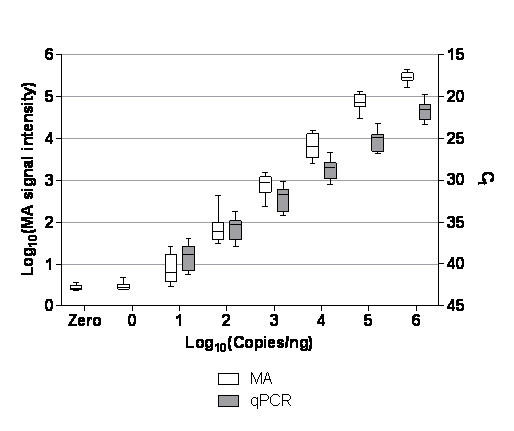
**Characterisation of platform signal output using RNA standards**. Signal output data for all eight standards across a tested range between 0 and 10^6 ^copies/ng total RNA are shown as box-whisker diagrams for microarray (white bars) and RT-qPCR (grey bars) applications. Median signal output (central line), interquartile range and minimum and maximum values are shown for each level of transcript abundance.

The signal output of each platform at different levels of copy number was measured in terms of microarray raw signal intensity or qPCR Ct values (Figure 1) for all eight RNA standards. Characterisation of the platform signal output for each level of RNA standard abundance may be a useful means of relating spike-in metrics to endogenous genes, as, unlike the copy number of *in vitro *produced RNA which can be ascertained by UV spectroscopy, the absolute copy numbers of endogenous transcripts are not normally known for microarray or RT-qPCR experiments. Therefore if a gene of interest falls within a given range of microarray signal intensity or Ct value, the performance characteristics in terms of the linearity, precision and accuracy of detection at that level of abundance can be used to inform these metrics for the candidate markers. Such platform-specific performance characteristics are further investigated in the following two sections.

### Linear range and LOD of microarray and RT-qPCR platforms

**Figure 2:**
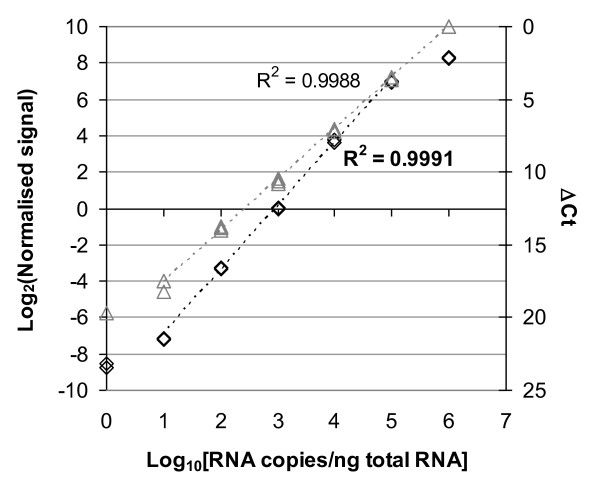
**Linear range of detection on microarray and RT-qPCR platforms**. Example plots of modelling the linear range of each gene expression platform are displayed. Normalised signal intensities (microarrays) (symbol: black diamond) or ΔCt values (RT-qPCR) (symbol: grey triangle) are plotted against the standard (copy number/ng). Individual data points for ERCC-13 from three independent experiments are plotted. Correlation analysis was performed over the linear portion of the detection range (Microarrays: 10-10^5 ^copies/ng; RT-qPCR 10 - 10^6 ^copies/ng) and Pearson correlation coefficient (R^2^) values are displayed (microarray value in bold).

The detection range of RNA standards was modelled across a range of copy numbers for each standard on both microarray and RT-qPCR platforms in order to define the linear dynamic range and LOD. Example plots of normalized microarray and RT-qPCR signal in correlation with the copy number are shown for ERCC-13 in Figure 2, with results for the other seven RNA standards presented in Additional File 2. Visual inspection of the results suggested that the linear region of the range was between 10 and 10^5 ^copies/ng RNA for Agilent microarrays and upwards of 10 copies/ng for the RT-qPCR platform. In order to confirm that the linear range of the instruments also corresponded to the dynamic range of signal output, the 10-fold differences in copy number between samples were compared with signal output to check for proportionality. Log_2_-transformations of the copy numbers for the standards were plotted against normalised signal outputs (microarray) or ΔCt values (RT-qPCR) and linear regression was performed across the linear range marked in Figure 2. The resulting slope and R^2 ^values from this analysis are displayed in Table 2. These results show that the expected slopes for all eight standards are close to the ideal value of 1.0 and the R^2 ^values indicate good correlation of data across the defined linear ranges (R^2 ^> 0.96, microarray; R^2 ^> 0.99, RT-qPCR).

**Table 2:** Accuracy and precision of platforms across linear range

	Platform			
**Standards**	**Microarray**		**RT-qPCR**	

	**Slope**	**R**^**2**^	**Slope**	**R**^**2**^

13	1.0566	0.9991	1.0004	0.9988

42	1.0047	0.9675	1.0205	0.9988

81	1.0389	0.9671	0.9946	0.9994

84	0.9772	0.9639	0.9693	0.9989

95	0.957	0.9712	1.0022	0.9993

99	1.0095	0.9626	1.0052	0.9994

113	1.0248	0.9993	1.0200	0.9970

171	1.0696	0.9993	0.9714	0.9988

The accuracy and precision of the platforms across the linear range (Microarray: 10-10^5 ^copies/ng; RT-qPCR: 10-10^6 ^copies/ng) were assessed by the slope (ideal slope = 1.000) and Pearson correlation coefficient (R^2^) values respectively, based on linear regression of transcript copies/ng *vs*. normalised signal intensity (microarray) or ΔCt values (RT-qPCR). RT-qPCR values were adjusted for the PCR efficiency of the assay.

**Figure 3:**
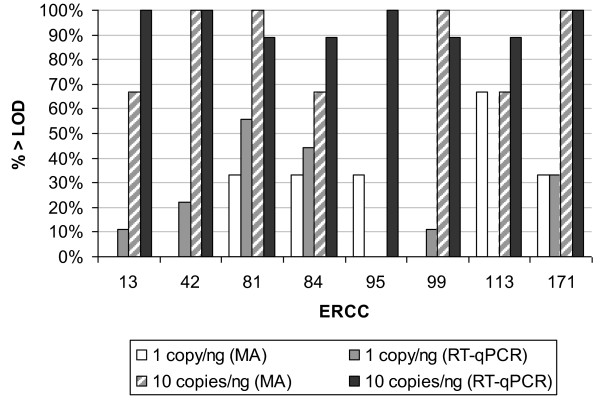
**LOD of ERCC standards on microarray and RT-qPCR platforms**. Percentage of assays exceeding the LOD for microarray (MA) and RT-qPCR platforms are displayed for each RNA standard for 1 copy/ng and 10 copies/ng total RNA abundance levels (Table 1), based on three replicate assays (microarrays) or nine replicate PCR assays (RT-qPCR). LOD is defined as the upper limit of the 95% confidence interval of 0 copies (microarrays) or positive Ct values (RT-qPCR).

The LOD of both platforms was also compared using data generated from all eight standards. For microarrays, the LOD was defined as the upper 95% confidence interval of the signal intensity of the baseline for the blank sample and the percentage of data points with signal output above the LOD for each copy number level calculated (Figure 3). For RT-qPCR, since the zero copy sample results in an 'undetermined' call, it is not possible to model a baseline signal level. Therefore the percentages of positive reactions were calculated for each level of abundance as the percentage above the LOD (Figure 3). At 1 copy/ng (equating to 1 RNA copy per qPCR reaction), it is evident that the standards could not be detected effectively on either microarray or RT-qPCR platforms as only 25% of microarray data exceeded the LOD, and 22% of PCR reactions resulted in a positive Ct value. When the copy number was increased to 10 copies/ng, 75% of microarray data exceed the LOD and 94% of qPCR reactions resulted in a Ct value. Therefore the LOD at which both platforms could discriminate between the presence and absence of the standards was estimated as 10 copies/ng. Similarly, at 100 copies/ng, the LOD was exceeded, with 100% of both microarray data and qPCR reactions yielding positive results based on the above criteria (data not shown).

### Technical reproducibility and precision of microarray and RT-qPCR platforms

Technical reproducibility and precision are another two important aspects of platform performance as knowledge of the technical 'noise' associated with the biomarker measurements is useful for informing the confidence with which results are interpreted, and assigning measurement uncertainty values for standardized assays. The standards were used to characterize the technical variation associated with each platform, both in terms of technical reproducibility (variation between experimental runs performed on different days) and precision (variation between replicate measurements performed within the same run).

**Figure 4:**
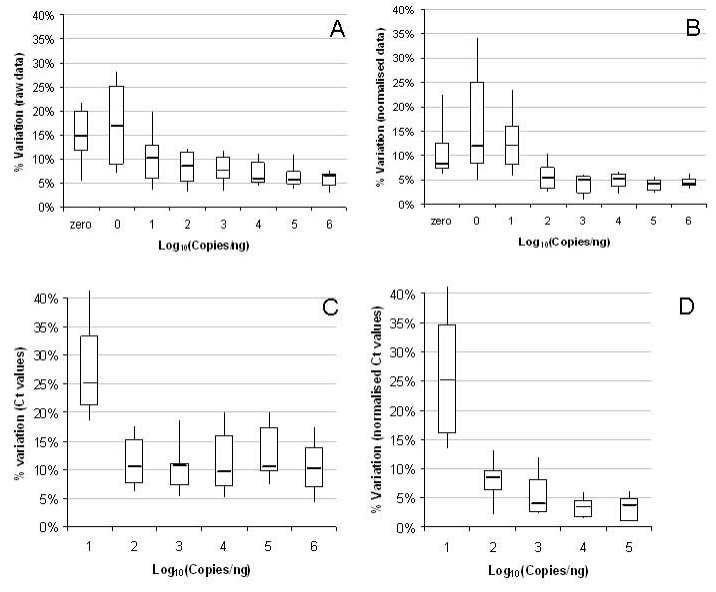
**Technical reproducibility of microarray and RT-qPCR platforms**. Technical reproducibility of microarray and RT-qPCR data was calculated based on the technical variation of each standard across three independent experiments using the same labelled sample (microarrays) or cDNA sample (RT-qPCR). Box-whisker plots show the median signal output (central line), interquartile range and 10^th ^and 90^th ^percentile values for each level of transcript abundance. Distribution of technical variation across all tested standards are displayed as percentage CV of microarray raw signal (SD/mean)(A), percentage variation associated with normalized signal intensity (log_2_) based on SD of normalized values (B), percentage variation in expression quantities (based on SD of Ct values, corrected for PCR efficiency of each assay)(C) and percentage variation in expression quantities, expressed relative to the mean Ct value for 10^6 ^copies/ng within each run (D).

The reproducibility of measurements from both gene expression platforms was calculated across a range of concentrations mimicking different transcript abundance levels (Figure 4). The variation in microarray measurements between different arrays performed on three separate occasions is displayed as raw or normalized signal intensities (Figure 4A and 4B respectively). It can be observed that technical reproducibility is poorer (median 10-20%) for low transcript abundance levels (1-10 copies/ng) which also happen to be at or below the LOD (see above section). The effect of normalization increases the spread of variation for the 1 copy/ng level, possibly due to calculation of expression values relative to the 75^th ^percentile (Figure 4B). However, above 100 copies/ng, the percentage variation of raw data is below 10%, and drops to 5% or less as a result of data normalisation (Figures 4A and 4B).

The technical reproducibility of microarray experiments across all probes (~44K) on the array, as opposed to the RNA standards only was also compared to determine if technical reproducibility of the standards correlated with the observed reproducibility for endogenous gene expression values. The results show that pair-wise correlation of replicate arrays of the same sample exhibited a high degree of correlation for the platform (R^2 ^> 0.985) (Additional File 3).

For RT-qPCR experiments, the data was compared between three different experimental runs (Figure 4C) based on the mean Ct value for each run. Due to the high number of failed reactions at 1 copy/ng (Figure 3), technical variation between runs was only calculated for greater than 10 copies/ng. Similar to microarray data, technical reproducibility was reduced at the low end of the linear detection range (Figure 2) reflecting a greater spread in Ct values. Above 100 copies/ng, technical variation between runs equated to 10%. Ordinarily, Ct values are not compared between runs since relative expression measurements are compared to a standard curve or reference sample incorporated on the same plate. Therefore Ct values were also expressed relative to the mean Ct value for the highest copy number (10^6 ^copies/ng) within each run in order to normalize for the effect of run-to-run variation. Transformation of RT-qPCR data to relative expression levels reduced the technical variation between runs to below the 5% level for copy numbers of 1000 or more (Figure 4D).

**Figure 5:**
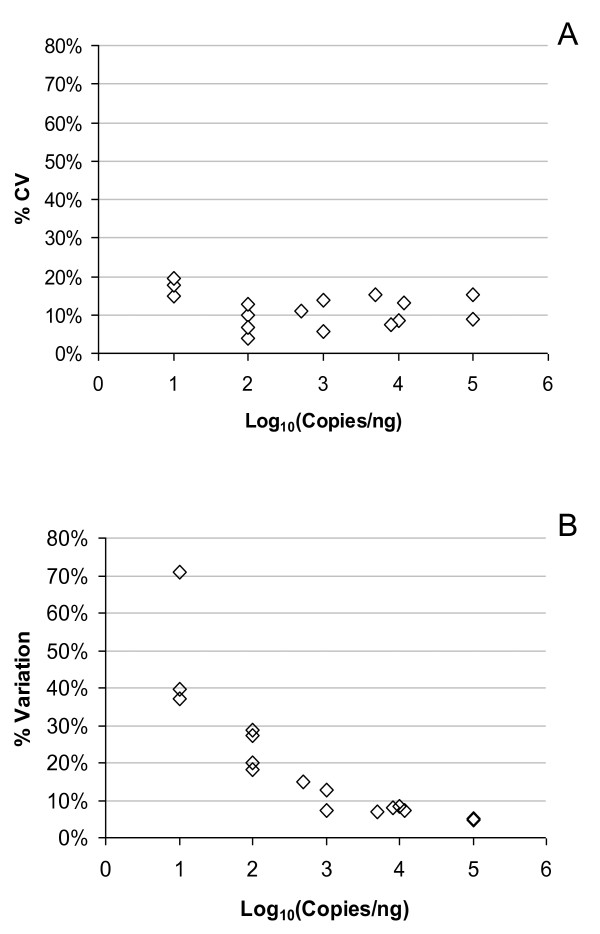
**Technical precision of replicate assays**. Precision of replicate measurements made within the same experimental run are plotted against copy number of the standards. Mean variation in raw signal intensities or Ct values across three independent runs are shown for microarray (A) and RT-qPCR (B), based on duplicate arrays or triplicate qPCR reactions within each run.

The technical precision of each platform, within the same hybridization experiment or qPCR plate, was also calculated for different copy number levels based on data from duplicate arrays or triplicate qPCR reactions (Figure 5). Results for within-run variation show that deviation in signal output was higher for both microarray and RT-qPCR platforms at the lower end of tested range (10 copies/ng). Within-run variation for microarrays was 10-20% above 100 copies/ng for all levels of abundance (Figure 5A), whereas higher technical reproducibility for RT-qPCR data correlated with increasing copy numbers (Figure 5B).

### Construction of 'normal' and 'disease' panels

Further to investigation of the applicability of RNA standards for provision of technical information on biomarker measurements, their usefulness for validating expression analyses of biomarkers between different conditions, e.g. normal *vs*. disease states was evaluated. Often the first stages of biomarker screening involves selecting the genes showing the largest and/or most significant fold changes in expression between different experimental groups, and studying the differences in global expression profiles using multifactorial analysis methods such as Principal Component Analysis (PCA) and ANOVA. For testing the utility of RNA standards in providing information on the success of discriminating different groups in the context of a trial or experiment, we constructed two panels (A and B) consisting of eight standards each, simulating normal and disease states (Table 3).

**Table 3:** Composition of 'normal' and 'disease' panels

RNA standards	Copies/ng total RNA		Fold change (B/A)
		
	Panel A	Panel B	
13	1 × 10^5^	1 × 10^5^	1.0

42	1 × 10^4^	5 × 10^3^	0.5

81	1 × 10^2^	1 × 10^2^	1.0

84	1 × 10^2^	5 × 10^2^	5.0

95	1 × 10^3^	1 × 10^3^	1.0

99	8 × 10^3^	1.2 × 10^4^	1.5

113	1 × 10^1^	1 × 10^1^	1.0

171	1 × 10^1^	1 × 10^2^	10.0

To mimic the composition of 'normal' and 'disease' states which contain both differentially expressed genes (DEGs) and non-DEGs, two panels were produced using the eight standards at defined ratios and copy numbers.

In order to mimic candidate biomarkers showing differential or unchanged expression in different experimental groupings, four of the standards exhibited an altered expression profile between the two panels, with ratios of 1.5, 2.0, 5.0 and 10.0 between groups, whilst the other four had a fold change of 1.0, with equal copy numbers in both panels (Table 3). A range of transcript abundance levels were included in the design of the panels, with the aim of mimicking, for example, transcripts with low abundance in the 'normal' state with a large increase in expression in the 'disease' states (e.g. ERCC-171) or transcripts of average abundance with a moderate fold change in the disease-state (e.g. ERCC-99) (Table 3). Three independent microarray experiments were set up for investigating the effect of variability due to labelling (duplicate reactions) and hybridisation (duplicate hybridisations per labelling reaction) on the discrimination of two panels.

### Accuracy of fold change estimation by microarray and RT-qPCR platforms

**Figure 6:**
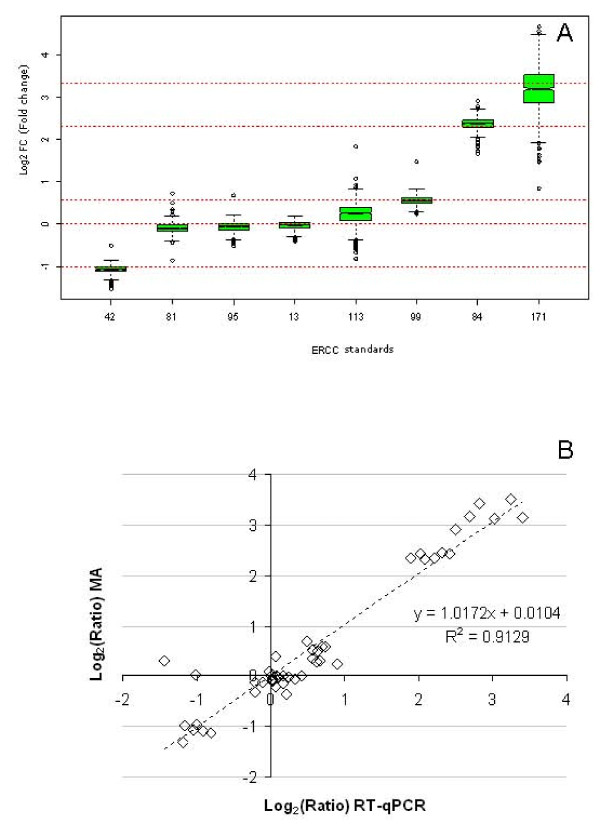
**Accuracy of fold change estimation**. The fold change in expression levels between panels A and B was calculated based on six pair-wise comparisons across three experimental runs. (A) The distribution of fold change measurements are displayed for each ERCC standard based on 100 individual microarray features for each standard. Box-whisker plots reflect median, interquartile range, 10^th ^and 90^th ^percentile fold change values with dots indicating individual outliers. Expected values for fold changes are indicated by gridlines. (B) Microarray observed fold change values averaged across all 100 probe replicates are plotted against fold change measurements from RT-qPCR data as individual data points (n = 6 for each ERCC standard). Trendline indicates correlation analysis with calculated slope and Pearson's correlation coefficient (R).

Initially the simulated 'normal' and 'disease' panels were used to assess the accuracy of fold change measurements using microarrays. Since 100 replicate probes specific for each of the RNA standards were present on the microarrays, they were treated as individual entities in order to model the distribution of fold change measurements for an individual feature, as is the case for most genes represented on whole genome microarrays. The accuracy of fold change estimation for each feature was assessed based on six pair-wise comparisons between the two panels, spanning the three independent experiments (Figure 6A). Overall the observed fold change measurements correlated closely to the expected values. The standards represented at medium to high abundance (10^3 ^copies/ng or above; ERCC-13, -42, -95 and -99) showed the closest agreement between observed and expected fold change values with over 75% of entities within 10% of the expected values. However, low abundance transcripts (10^2 ^copies/ng or equivalent to 1 copy/cell) in the panels (ERCC-81 and -84), resulted in just over 60% entities falling within the same threshold, whilst trace abundance transcripts (10^1 ^copies/ng; ERCC-113 and -171), exhibited significantly impaired accuracy with less than 25% of entities falling within 10% of the expected values. As real-time PCR is the main strategy used for validation of microarray fold change measurements [22], the microarray fold change data (averaged over probe replicates) was compared with RT-qPCR measurements of the panels. Similarly, replicate independent RT and qPCR experiments were performed in order to encapsulate full run-to-run variation for the technique. Good agreement between the two platforms was shown by correlation analysis of the fold change measurements for microarray and RT-qPCR platforms (R = 0.96) (Figure 6B).

### Classification of differentially expressed genes

**Figure 7:**
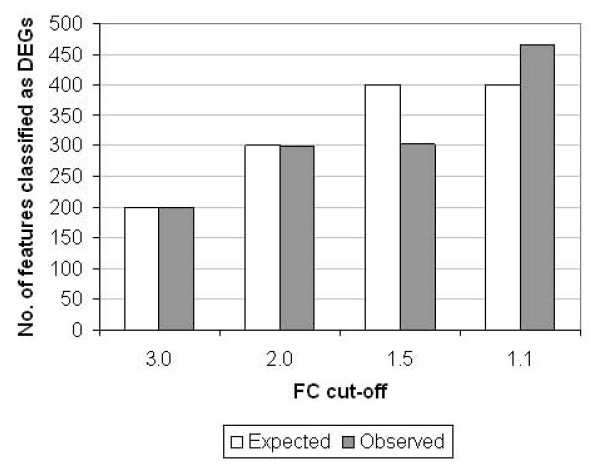
**Classification of DEGs**. The expected and observed number of ERCC microarray features classified as DEGs are compared using fold change cut-offs of 3.0, 2.0, 1.5 and 1.1. A statistical cut-off of *p *< 0.05 was applied. Analysis was based on averaged data for 'normal' and 'disease' panels A and B (n = 6) with 100 microarray features present for each ERCC standard.

Fold change cut-offs and statistical analysis are the most commonly used approaches for generating gene lists which are further interrogated in terms of biological significance by gene ontology and pathway analysis [23]. We investigated whether individual features (each array contained 100 replicates for each standard) were classified correctly using a 'Volcano plot' approach with fold change cut-off thresholds of 3.0, 2.0, 1.5 and 1.1 with a *p*-value cut-off of 0.05. Figure 7 shows that using the higher fold change cut-off thresholds of 3.0 and 2.0, almost 100% of features are classified correctly as differentially (ERCC-42, -84 and -171) or non-differentially expressed (ERCC-13, -81, -95, -99 and -113). However using a fold change cut-off threshold of 1.5, only 2% of ERCC-99 features are correctly classified as differentially expressed despite being spiked at a 1.5-fold ratio in the two panels. Using the low stringency 1.1-fold cut-off, some features are misclassified as exhibiting differential expression despite their presence at the same level in both panels. Of the 65 incorrectly classified 'false positive' features, 48 were probes detecting ERCC-113 and 17 were those detecting ERCC-81, the two standards with the lowest abundance of the four unchanged standards between the panels.

### Discrimination between 'normal' *vs*. 'disease' panels

**Figure 8:**
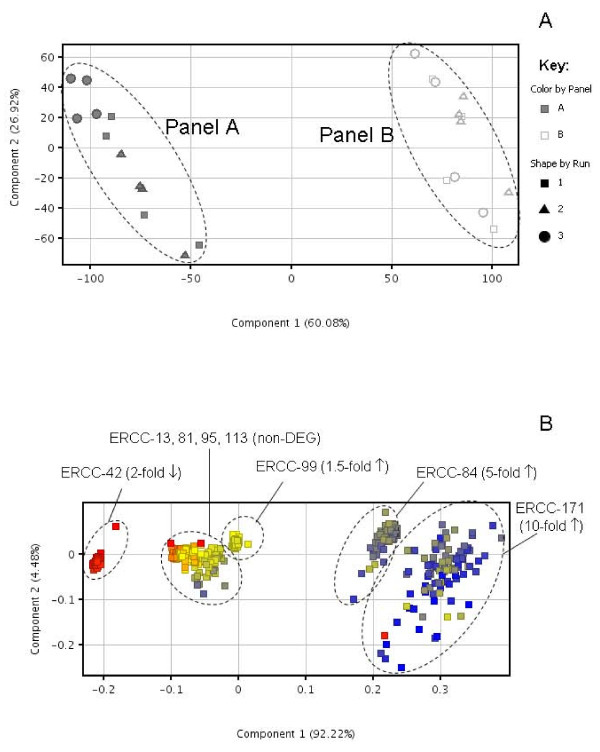
**Discrimination between expression profiles of ERCC microarray data**. The discrimination between ERCC 'normal' and 'disease' panels A and B across three independent microarray experiments (Runs 1, 2, 3) was assessed in the following ways: (A) PCA was performed using expression data for all eight ERCC standards containing 100 replicates of each probe based on conditions (panel and experimental run). (B) Discrimination of microarray measurements between DEGs and non-DEGs was assessed by PCA based on entities. Dots represent individual microarray features (100 per standard).

Further to the application of the panels to fold change analysis, their usefulness for assessing the robustness of discriminating between normal and diseased states, using multigene expression profiles, was also investigated. The impact of technical factors such as target labelling and microarray hybridization on the discrimination between the two simulated 'normal' and 'disease' panels was investigated by PCA of the microarray data and the results are presented in Figure 8A. The two panels, A and B, are separated clearly by the first principal component, which accounts for the majority (60%) of the variation in expression profiles. The second principal component accounts for a further 27% of the variation in the data which may be surmised to be due to technical 'noise' in the experimental system (since panel and experimental run were the only variables in the experiment). Experimental run does not appear to have a systematic effect on the expression profile of the RNA standards, as individual runs do not cluster together on the PCA (Figure 8A). The discrimination of the microarray data based on entities (genes) with a similar expression profile was also investigated using PCA (Figure 8B). The analysis indicates clear discrimination between RNA standards with different ratios between the panels, with over 90% of the variation in expression profile being due to this factor. Standards, whose expression is 'up-regulated' in panel B compared to panel A, are clearly separated from those with no fold difference between panels or down-regulated in panel B. ERCC-99, which exhibited only a 1.5-fold difference between panels, clustered close to the four standards with unchanged expression levels between panels (Figure 8B). The second principal component, which could be attributable to technical variation, accounts for only 5% in the variation (in expression of individual standards) and shows highest spread for ERCC-171 which has a ratio of 10.0 between panels A and B. As noted for accuracy of fold change estimation for different standards, the expression of ERCC-171 may be more variable due to a higher level of technical noise in the region of lower transcript abundance (Figure 6A).

## Discussion

The complexity and multivariate nature of gene expression profiling techniques, measuring thousands of different transcripts has raised issues over the last decade regarding suitable approaches for standardized data comparison. In addition to the different techniques available (microarray, RT-qPCR, next generation sequencing etc), factors such as platform, laboratory, experimental run, experimental design and methodological parameters such as labelling approach for microarray studies or choice of RT priming strategy RT-qPCR experiments can all impact on the results [24-26]. Furthermore, processing and normalization of the results set can also influence both microarray and RT-qPCR data [27,28]. Significant progress, in improving the confidence in the extent to which technical variability influences results from different microarray platforms, has been made through the application of reference RNA sample titrations by the MAQC [29]. However, as microarray and other transcriptomic approaches move from being predominantly research tools into clinical and regulatory applications, reference materials are required in order to ensure high levels of quality control and traceable results [30]. The development of generic RNA standards by the ERCC are expected to fulfil such function as reference materials [19]. In this study we have sought to demonstrate how carefully constructed panels of RNA standards can be used to characterize platform-associated technical factors and provide information relevant to biomarker discovery and validation.

For this study, technical aspects of platforms performance were investigated using eight different RNA standards, mimicking transcripts covering a wide physiological range of abundance levels. By varying each standard across the full range of copy numbers, some of the confounding influence caused by individual microarray probe specificity, which is present when a single spike-in is used at a set concentration, is negated [20]. Likewise, variations due to Taqman assay efficiency are likely to be minimised by performing RT-qPCR reactions for a panel of multiple standards.

The linear range of both microarray and RT-qPCR platforms was assessed by the slope of signal output *vs*. spike-in concentration and by characterizing the lower LOD using the standards. The dynamic range of detection using the Agilent one-colour platform encompassed four orders of magnitude over which the observed changes in signal intensity closely matched the expected values (Figure 2A). This result conforms with product literature for the platform, reporting a five order of magnitude detection range (Agilent Multiplex Gene Expression Microarrays product information, Part No. 5989-5432EN). At concentrations of transcripts 10-fold below the LOD range, the signal intensities were generally indistinguishable from non-specific background hybridisation data (Figure 3). Results of RT-qPCR analysis of the same panels displayed linearity over the same range of copy numbers as the microarray results. However no plateau was observed at the maximum copy number tested (10^6 ^copies per ng) (Figure 2B), in keeping with the dynamic range of seven to eight orders of magnitude reported for real-time PCR [31]. The LOD for RT-qPCR was in the region of 10 copies (Figure 3), which is close to the reported sensitivity of RT-qPCR for single copy detection [32] in view of the fact that the reverse transcription step is normally significantly less than 100% efficient [33]. Concentration response curves using RNA standards have previously been shown to be a useful tool for comparing the linearity of the response of different microarray platforms in terms of signal compression and precision [12]. It is proposed that panels of universally applicable standards, such as those used here, will provide directly comparable information on the performance of different platforms in terms of dynamic range and LOD.

The panels also yielded useful information on the reproducibility of the two gene expression technologies investigated. For transcript copy numbers of at least 100 copies per ng total RNA (approximately 1 copy per cell), technical reproducibility of microarray data (Figure 4A) was within the 5-15% CV values for the Agilent one-colour platform reported by the MAQC [29]. For lower abundance transcripts, reproducibility between experimental runs was poorer with variation of up to 30-35% observed for raw and normalized microarray data (Figure 4). For genes expressed at such low levels, stringent filtering of the data based on present/absent calls or raw expression levels has been shown to improve concordance between replicate arrays [34,35]. Likewise, for RT-qPCR, increased inter-run and intra-run variation was observed for low abundance transcripts (Figures 4 and 5) due to stochastic variation in both RT and qPCR stages when only a small number of molecules is present in each reaction [17]. The RNA standards can therefore provide an indication of the measurement uncertainty associated with biomarkers of varying abundance and could be used to develop guidelines, e.g. for trace biomarker measurements and for calculating the number of replicate measurements required to ensure sufficient statistical power in a particular experiment or assay.

In addition to their utility for cross-platform comparison and measurement uncertainty considerations, we sought to demonstrate that RNA standards can also facilitate internal quality control of biomarker measurements in terms of differential expression analysis and multigene expression profiling techniques. It is envisaged that such panels could be spiked into experimental samples in order to gauge the accuracy with which 'normal' and 'disease' states or control and treatment conditions are assigned.

By using the RNA standards as surrogate biomarkers, the panels were tested to gauge the confidence of assigning differential or non-differential expression of a biomarker at particular levels of transcript abundance or magnitude of fold change (Figure 6). The observed fold changes highlighted the variable extent to which technical noise arising from microarray labelling and hybridisation may impact on biomarker regulation. Transcripts of lower abundance showed wider variation in fold change measurements for both unchanged and differential ratios between the two panels. The distribution of fold change ratios between the panels modelled on the 100 probe replicates on each array (Figure 6A) indicate that erroneous fold change results are more likely to arise in the region corresponding to lower signal intensity. Comparison of microarray and RT-qPCR results revealed good consistency between the two technologies for fold change detection (Figure 6B) and also confirmed that the Agilent platform does not cause compression of fold change measurements [29]. The panels of standards provide further opportunities for QC of gene expression results when applying different analytical methods, such as fold change cut-off thresholds and statistical testing. For example, our results highlighted that biomarkers, with a 1.5-fold change between experimental groups, are less easily discriminated than at higher fold changes (≥ 2.0-fold) (Figures 7 and 8B).

The discrimination between the 'normal' and 'disease' panels using global profiling methods such as PCA (Figure 8) was also shown to be a potentially useful QC tool for investigating technical noise within an experiment and could be employed for identifying anomalous microarrays within a dataset. Such analyses of the panels also indicate whether run-to-run variation has greater impact on the dataset compared to true differences in conditions (e.g. normal *vs*. disease) or between entities, i.e. Differentially Expressed Genes (DEG) and non-DEG. For the Agilent microarray platform used in this study, experimental runs did not have a systematic effect on the profile of the eight RNA standards. Also, individual sample labelling using a one-colour approach did not seem to cause any observable bias in the resultant expression profiling (Figure 8). However, it has been reported that two-colour sample labelling had a significant effect on the gene expression profile, especially between different laboratories [36].

## Conclusions

RNA standards provide a means of internal quality control for all stages of the gene expression experiment, namely sample processing, assay methodology, data processing and analysis. In this study, we have demonstrated that panels of generic RNA standards can be used to assess inter-platform variations in terms of dynamic range, LOD and precision of different technologies. We found that Agilent one-colour microarray hybridisation data and RT-qPCR measurements both provided accurate and reproducible measurement of the standards, although transcript abundance has a significant influence on these parameters. Furthermore, simulated 'normal' and 'disease' panels proved to be informative for the analysis of fold change accuracy and the discrimination of transcriptomic measurements. We conclude that such prototype reference panels could be useful QC materials for the standardization of gene expression measurements between laboratories and platforms, and in aiding interpretation of biomarker profiling data in regulatory settings.

## Methods

### Preparation of *in vitro *transcribed RNA and samples

*In vitro *transcribed RNA standards were produced from original ERCC plasmid DNA (donated by Dr. Marc Salit, NIST, USA). Plasmid DNA from ERCC standards 13, 42, 81, 84, 95, 99, 113 and 171 (for sequence information, see Additional File 1) were cleaved into a single linear strand using BamH*I *restriction endonuclease enzyme (New England Biolabs, Hitchin, UK). *In vitro *transcription was performed using Ambion MEGAscript^® ^T7 Kit (Applied Biosystems, Warrington, U.K.) followed by DNase treatment and clean-up using RNeasy columns (Qiagen, Hilden, Germany). RNA concentration and length were measured using Nanodrop 1000 spectrophotometer (Thermo Scientific, Wilmington, DE) and 2100 Bioanalyzer system (Agilent Technologies, Waldbronn, Germany) respectively. RNA stocks were diluted in nuclease free-water and spiked into Universal Human Reference RNA (Stratagene, U.K.) at varying concentrations and ratios (Tables 1 and 3).

### Microarray labelling and hybridization

RNA labelling and hybridization were performed according to the Agilent One-Colour Microarray-Based Gene Expression Analysis (Quick Amp Labelling) protocol (v5.7 March 2008). 500 ng of total RNA was labelled with cyanine-3 and assessed for yield and dye incorporation using the Nanodrop spectrophotometer. Purified cRNA samples were hybridized to custom-made Agilent 4 × 44 K oligo microarray. The array design incorporated 100 replicate probes for each RNA standard (Additional File 1) in the background of human genome reference sequences. Microarray slides were scanned at 5 μM resolution using an extended dynamic range (XDR) scan at 10% and 100% PMT gain on Agilent G2505B scanner. Feature extraction was performed using Agilent Feature Extraction software v9.5.1.1.

### Microarray data processing and analysis

Microarray data analysis was performed using GeneSpring GX 11.0 software (Agilent Technologies). Green processed signal (gProcessed Signal) was selected as background corrected signal and normalized to the 75th percentile for each feature and baselined to median of all samples. Data for linear range, LOD, technical reproducibility and precision assessments were calculated using the median value of 100 replicate features for each RNA standard within each microarray. Median-averaged values for each microarray were further analysed in Microsoft Excel (2003). PCA was performed using an entity list comprising of features for the eight standards only (100 features for each). Fold change analysis was carried out with normalized data for each individual feature averaged across two replicate microarrays using 'R' software [37]. Fold changes between the two panels were calculated based on six pairwise comparisons between labelled samples for panels 'A' and 'B' within each microarray run. PCA was performed in GeneSpring GX 11.0 using four principal components and was mean-centred and scaled.

### RT-qPCR analysis

RT-qPCR was performed using a two-step protocol. RNA samples were reverse-transcribed using the Taqman Reverse Transcription Reagents Kit (Applied Biosystems, Foster City, CA) in 40 μl reactions containing 400 ng total RNA with oligo(dT) primers according to the manufacturer's instructions. cDNA samples were diluted to a concentration of 0.5 ng/μl (total RNA equivalent) with nuclease-free water. 20 μl qPCRs containing 1 ng RNA equivalent were performed in optical 96-well plates using an ABI PRISM^® ^7900HT Sequence Detection System (Applied Biosystems, Warrington, U.K.). Custom-designed primers and Taqman FAM-TAMRA probes (Additional File 1) were supplied by Applied Biosystems. PCR efficiencies for each assay were calculated based on a separate experiment with a serial dilution of pure ERCC cDNA (in the absence of background RNA). PCRs were prepared using TaqMan^® ^Universal PCR Master Mix with a final primer concentration of 900 nM and probe concentration of 250 nM. The following reaction conditions were used: 50°C for 2 minutes, 95°C for 10 minutes, followed by 45 cycles of 95°C for 15 seconds and 60°C for 60 seconds. Triplicate reactions were set up for each sample. Baseline fluorescence was set automatically and the quantification threshold (C_t_) was set manually to the same level for all runs within an experiment. Data was analysed in Microsoft Excel (2003). For Experiment 1, ΔCt values were calculated relative to the mean value for the 10^6 ^copies/ng of the RNA standard within each run (plate). For Experiment 2 ('normal' and 'disease' panels), ΔCt values were calculated by pairwise comparison of the mean Ct value for a panel A sample *vs*. that of a panel B sample.

## Authors' contributions

AD performed microarray and RT-qPCR experiments, participated in the design of the study, performed data analysis and drafted the manuscript. RE designed custom microarrays, supervised experiments, participated in the design of the study and helped to draft the manuscript. CF conceived of the study, and participated in its design and coordination and helped to draft the manuscript. All authors read and approved the final manuscript.

## Supplementary Material

Additional file 1 **Information about RNA sequences and RT-qPCR assays**. This Microsoft Word file gives details of ERCC RNA sequences, microarray probes and Taqman^® ^assays. Click here for file

Additional file 2 **Linear range of detection on microarray and RT-qPCR platforms**. This Microsoft Powerpoint file presents data related to correlation of transcript copy number with normalized microarray signal output and RT-qPCR ΔCt values for all standards investigated. Click here for file

Additional file 3 **Results of correlation analysis of whole array data**. Microsoft Word file displays data from pairwise correlation of microarray data (all entities) from three replicate microarrays. Click here for file

## References

[B1] Waring JF Halbert DN The promise of toxicogenomics Curr Opin Mol Ther 2002 4 229 235 12139308

[B2] Bhattacharya S Mariani TJ Array of hope: expression profiling identifies disease biomarkers and mechanism Biochem Soc Trans 2009 37 855 862 10.1042/BST0370855 19614607

[B3] Bustin SA Dorudi S Gene expression profiling for molecular staging and prognosis prediction in colorectal cancer Expert Rev Mol Diagn 2004 4 599 607 10.1586/14737159.4.5.599 15347254

[B4] Ross JS Hatzis C Symmans WF Pusztai L Hortobagyi GN Commercialized multigene predictors of clinical outcome for breast cancer Oncologist 2008 13 477 493 10.1634/theoncologist.2007-0248 18515733

[B5] Buyse M Loi S van't Veer L Viale G Delorenzi M Glas AM Saghatchian d'Assignies M Bergh J Lidereau R Ellis P Validation and Clinical Utility of a 70-Gene Prognostic Signature for Women With Node-Negative Breast Cancer J Natl Cancer Inst 2006 98 1183 1192 10.1093/jnci/djj329 16954471

[B6] Paik S Shak S Tang G Kim C Baker J Cronin M Baehner FL Walker MG Watson D Park T A multigene assay to predict recurrence of tamoxifen-treated, node-negative breast cancer N Engl J Med 2004 351 2817 2826 10.1056/NEJMoa041588 15591335

[B7] FDA Critical Path Opportunities List 2006 http://www.fda.gov/downloads/ScienceResearch/SpecialTopics/CriticalPathInitiative/CriticalPathOpportunitiesReports/UCM077258.pdf

[B8] Cahan P Rovegno F Mooney D Newman JC St LG III McCaffrey TA Meta-analysis of microarray results: challenges, opportunities, and recommendations for standardization Gene 2007 401 12 18 10.1016/j.gene.2007.06.016 17651921 PMC2111172

[B9] Kawasaki ES The end of the microarray Tower of Babel: will universal standards lead the way? J Biomol Tech 2006 17 200 206 16870711 PMC2291790

[B10] Cronin M Ghosh K Sistare F Quackenbush J Vilker V O'Connell C Universal RNA Reference Materials for Gene Expression Clin Chem 2004 50 1464 1471 10.1373/clinchem.2004.035675 15155546

[B11] Andersen MT Foy CA The development of microarray standards Anal Bioanal Chem 2005 381 87 89 10.1007/s00216-004-2825-5 15614502

[B12] Tong W Lucas AB Shippy R Fan X Fang H Hong H Orr MS Chu TM Guo X Collins PJ Evaluation of external RNA controls for the assessment of microarray performance Nat Biotechnol 2006 24 1132 1139 10.1038/nbt1237 16964227

[B13] Huggett J Dheda K Bustin S Zumla A Real-time RT-PCR normalisation; strategies and considerations Genes Immun 2005 6 279 284 10.1038/sj.gene.6364190 15815687

[B14] Thellin O Zorzi W Lakaye B De Borman B Coumans B Hennen G Grisar T Igout A Heinen E Housekeeping genes as internal standards: use and limits J Biotechnol 1999 75 291 295 10.1016/S0168-1656(99)00163-7 10617337

[B15] Vandesompele J De Preter K Pattyn F Poppe B Van Roy N De Paepe A Speleman F Accurate normalization of real-time quantitative RT-PCR data by geometric averaging of multiple internal control genes Genome Biol 2002 3 RESEARCH0034 10.1186/gb-2002-3-7-research0034 12184808 PMC126239

[B16] Gilsbach R Kouta M Bonisch H Bruss M Comparison of *in vitro *and *in vivo *reference genes for internal standardization of real-time PCR data Biotechniques 2006 40 173 177 10.2144/000112052 16526406

[B17] Bustin SA Nolan T Pitfalls of quantitative real-time reverse-transcription polymerase chain reaction J Biomol Tech 2004 15 155 166 15331581 PMC2291693

[B18] Baker SC Bauer SR Beyer RP Brenton JD Bromley B Burrill J Causton H Conley MP Elespuru R Fero M The External RNA Controls Consortium: a progress report Nat Methods 2005 2 731 734 10.1038/nmeth1005-731 16179916

[B19] External RNA Controls Consortium Proposed methods for testing and selecting the ERCC external RNA controls BMC Genomics 2005 6 150 10.1186/1471-2164-6-150 16266432 PMC1325234

[B20] McCall MN Irizarry RA Consolidated strategy for the analysis of microarray spike-in data Nucleic Acids Res 2008 36 e108 10.1093/nar/gkn430 18676452 PMC2553586

[B21] Clinical and Laboratory Standards Institute Use of External RNA Controls in Gene Expression Assays; Approved Guideline MM16-A 2006 26 29

[B22] Canales RD Luo Y Willey JC Austermiller B Barbacioru CC Boysen C Hunkapiller K Jensen RV Knight CR Lee KY Evaluation of DNA microarray results with quantitative gene expression platforms Nat Biotechnol 2006 24 1115 1122 10.1038/nbt1236 16964225

[B23] Guo L Lobenhofer EK Wang C Shippy R Harris SC Zhang L Mei N Chen T Herman D Goodsaid FM Hurban P Rat toxicogenomic study reveals analytical consistency across microarray platforms Nat Biotechnol 2006 24 1162 1169 10.1038/nbt1238 17061323

[B24] Bammler T Beyer RP Bhattacharya S Boorman GA Boyles A Bradford BU Bumgarner RE Bushel PR Chaturvedi K Choi D Standardizing global gene expression analysis between laboratories and across platforms Nat Methods 2005 2 351 356 10.1038/nmeth0605-477a 15846362

[B25] Irizarry RA Warren D Spencer F Kim IF Biswal S Frank BC Gabrielson E Garcia JG Geoghegan J Germino G Multiple-laboratory comparison of microarray platforms Nat Methods 2005 2 345 350 10.1038/nmeth756 15846361

[B26] Stahlberg A Hakansson J Xian X Semb H Kubista M Properties of the reverse transcription reaction in mRNA quantification Clin Chem 2004 50 509 515 10.1373/clinchem.2003.026161 14726469

[B27] Harr B Schlotterer C Comparison of algorithms for the analysis of Affymetrix microarray data as evaluated by co-expression of genes in known operons Nucleic Acids Res 2006 34 e8 10.1093/nar/gnj010 16432259 PMC1345700

[B28] Cikos S Bukovska A Koppel J Relative quantification of mRNA: comparison of methods currently used for real-time PCR data analysis BMC Mol Biol 2007 8 113 10.1186/1471-2199-8-113 18093344 PMC2235892

[B29] Shi L Reid LH Jones WD Shippy R Warrington JA Baker SC Collins PJ de Longueville F Kawasaki ES Lee KY The MicroArray Quality Control (MAQC) project shows inter- and intraplatform reproducibility of gene expression measurements Nat Biotechnol 2006 24 1151 1161 10.1038/nbt1239 16964229 PMC3272078

[B30] Shi L Perkins RG Fang H Tong W Reproducible and reliable microarray results through quality control: good laboratory proficiency and appropriate data analysis practices are essential Curr Opin Biotechnol 2008 19 10 18 10.1016/j.copbio.2007.11.003 18155896

[B31] Wong ML Medrano JF Real-time PCR for mRNA quantitation Biotechniques 2005 39 75 85 10.2144/05391RV01 16060372

[B32] Palmer S Wiegand AP Maldarelli F Bazmi H Mican JM Polis M Dewar RL Planta A Liu S Metcalf JA New real-time reverse transcriptase-initiated PCR assay with single-copy sensitivity for human immunodeficiency virus type 1 RNA in plasma J Clin Microbiol 2003 41 4531 4536 10.1128/JCM.41.10.4531-4536.2003 14532178 PMC254331

[B33] Levesque-Sergerie JP Duquette M Thibault C Delbecchi L Bissonnette N Detection limits of several commercial reverse transcriptase enzymes: impact on the low- and high-abundance transcript levels assessed by quantitative RT-PCR BMC Mol Biol 2007 8 93 10.1186/1471-2199-8-93 17953766 PMC2151766

[B34] Shippy R Sendera TJ Lockner R Palaniappan C Kaysser-Kranich T Watts G Alsobrook J Performance evaluation of commercial short-oligonucleotide microarrays and the impact of noise in making cross-platform correlations BMC Genomics 2004 5 61 10.1186/1471-2164-5-61 15345031 PMC517929

[B35] Shi L Tong W Fang H Scherf U Han J Puri RK Frueh FW Goodsaid FM Guo L Su Z Cross-platform comparability of microarray technology: intra-platform consistency and appropriate data analysis procedures are essential BMC Bioinformatics 2005 6 Suppl 2 S12 10.1186/1471-2105-6-S2-S12 16026597 PMC1637032

[B36] Ach RA Floore A Curry B Lazar V Glas AM Pover R Tsalenko A Ripoche H Cardoso F d'Assignies MS Robust interlaboratory reproducibility of a gene expression signature measurement consistent with the needs of a new generation of diagnostic tools BMC Genomics 2007 8 148 10.1186/1471-2164-8-148 17553173 PMC1904205

[B37] R Development Core Team R: A language and environment for statistical computing 2008 R Foundation for Statistical Computing, Vienna, Austria http://www.R-project.org ISBN 3-900051-07-0

