# Supplementary material for: Evaluation of external RNA controls for the standardisation of gene expression biomarker measurements
Source: BMC Genomics. 2010 Nov 24;11:662. doi: 10.1186/1471-2164-11-662 (PMC3091780; doi:10.1186/1471-2164-11-662)
Supplement: Additional file 1 — Information about RNA sequences and RT-qPCR assays. This Microsoft Word file gives details of ERCC RNA sequences, microarray probes and Taqman® assays. [file 1471-2164-11-662-S1.DOC]

### Additional file 1 –ERCC RNA standard sequence information and sequences of ERCC microarray probes and Taqman assays

**Additional Table 1: RNA s**tandard sequence information

| **ERCC standardid.** | **GenBank accession** | **Length**  **(excl. polyA tail)** | **GC content (%)** |
| --- | --- | --- | --- |
| **ERCC-00013-01** | EF011062 | 784 | 42.8 |
| **ERCC-00042-01** | DQ516783 | 999 | 39.2 |
| **ERCC-00081-02** | DQ854991 | 509 | 48.7 |
| **ERCC-00084-01** | DQ883682 | 970 | 50.3 |
| **ERCC-00095-01** | DQ516759 | 495 | 37.2 |
| **ERCC-00099-01** | DQ875387 | 1324 | 41.3 |
| **ERCC-00113-01** | DQ883663 | 820 | 50.7 |
| **ERCC-00171-01** | DQ854994 | 481 | 47.7 |

**Additional Table 2: Microarray probe sequences for RNA standards**

| **Probe name** | **Sequence (5' - 3')** |
| --- | --- |
| **ERCC-013** | GGGAGTATGTCGGACATGGTGTTGGTCAAGACTTGCATGAGGACCCGCAA  TTCCTCATT |
| **ERCC-042** | CCTCCTTCACCAGTTCCCATGAATGTTCCACATTCTTTAACTGCCTTAGCAA  TGATAGG |
| **ERCC-081** | GCGTGATGGGATGAACGTCGCTTAAACGTTGGTTCAGGGCTCCATATATAC  TCTGCCGT |
| **ERCC-084** | GCTTCTAACTCGCTGTGAATCTACTGCAGAACTATGGGTTTGCTAGCGCGC  GGTATCTA |
| **ERCC-095** | GCAGTTCATCTTTAACCTCATCCCACAAAGCCGCTTTCTTTAAAGCCCACTCAA  AATCT |
| **ERCC-099** | CCCGAGCTGAATATCGGGGTCATTGCCAATGATATGGCAGCTTCTACATACGAATTAAAA |
| **ERCC-113** | GGAGAGAGAGGTTAATAAGGCCTAGCCTAAAGGTTCTTGCAGAGCAACATCATATACCCT |
| **ERCC-171** | GAAAACTGCGACTGTTCTTTAACCAAACATCCGTGCGATTCGTGCCACTCGTAGACGGCA |

Additional Table 3: Taqman assays

| **Standard id.** | **Forward primer** | **Reverse primer** | **Taqman® probe** | **Ampli-con size (bp)** |
| --- | --- | --- | --- | --- |
| **13** | CGGACATGGTGTTGGTCAAG | TTGTTGGGCGGACCGTAA | TGCATGAGGACCCGCAAATTCCTC | 66 |
| **42** | AGAGAGCTTTTGGCAATCCT | TCATTTGCTAAGGCAGTTAAAGA | TCACCAGTTCCCATGAATGTTCCAC | 73 |
| **81** | TGACGGCTTCAACGTTTTCA | TCTGATGTACCAGCGTGCAACT | TTTCCGCAGGTGGCGACCCTC | 65 |
| **84** | TGGATAAGCGAGGTCAGTCAAG | ATGCAGGCAAACGATCTACGT | ATTCGTTGCCTCCGGGTCC | 65 |
| **95** | GAGCGTTTTTATGCAGTTCATCTTT | GGATAAGATTGTTGAGTGGGCTTT | ACCTCATCCCACAAAGCCGCTTTCTT | 77 |
| **99** | TCGTCCATCCCTCAAGAGAGA | CGCAATCGCGTGTGAATG | CATGGAAAGAGCTCGACAAAATTTACTC | 71 |
| **113** | GCGACACCAACATCGTTACG | CCGCGCGTGAGCACTT | ACACACCGGACGCTTGGATCAGTG | 65 |
| **171** | TTAGTTTCGTGGCGGGATTT | CACGAATCGCACGGATGTT | AGGAAAACTGCGACTGTTCTTTAACC | 67 |
