# Supplementary material for: Evaluation of external RNA controls for the standardisation of gene expression biomarker measurements
Source: BMC Genomics. 2010 Nov 24;11:662. doi: 10.1186/1471-2164-11-662 (PMC3091780; doi:10.1186/1471-2164-11-662)
Supplement: Additional file 2 — Linear range of detection on microarray and RT-qPCR platforms. This Microsoft Powerpoint file presents data related to correlation of transcript copy number with normalized microarray signal output and RT-qPCR ΔCt values for all standards investigated. [file 1471-2164-11-662-S2.PPT]

## Slide 1
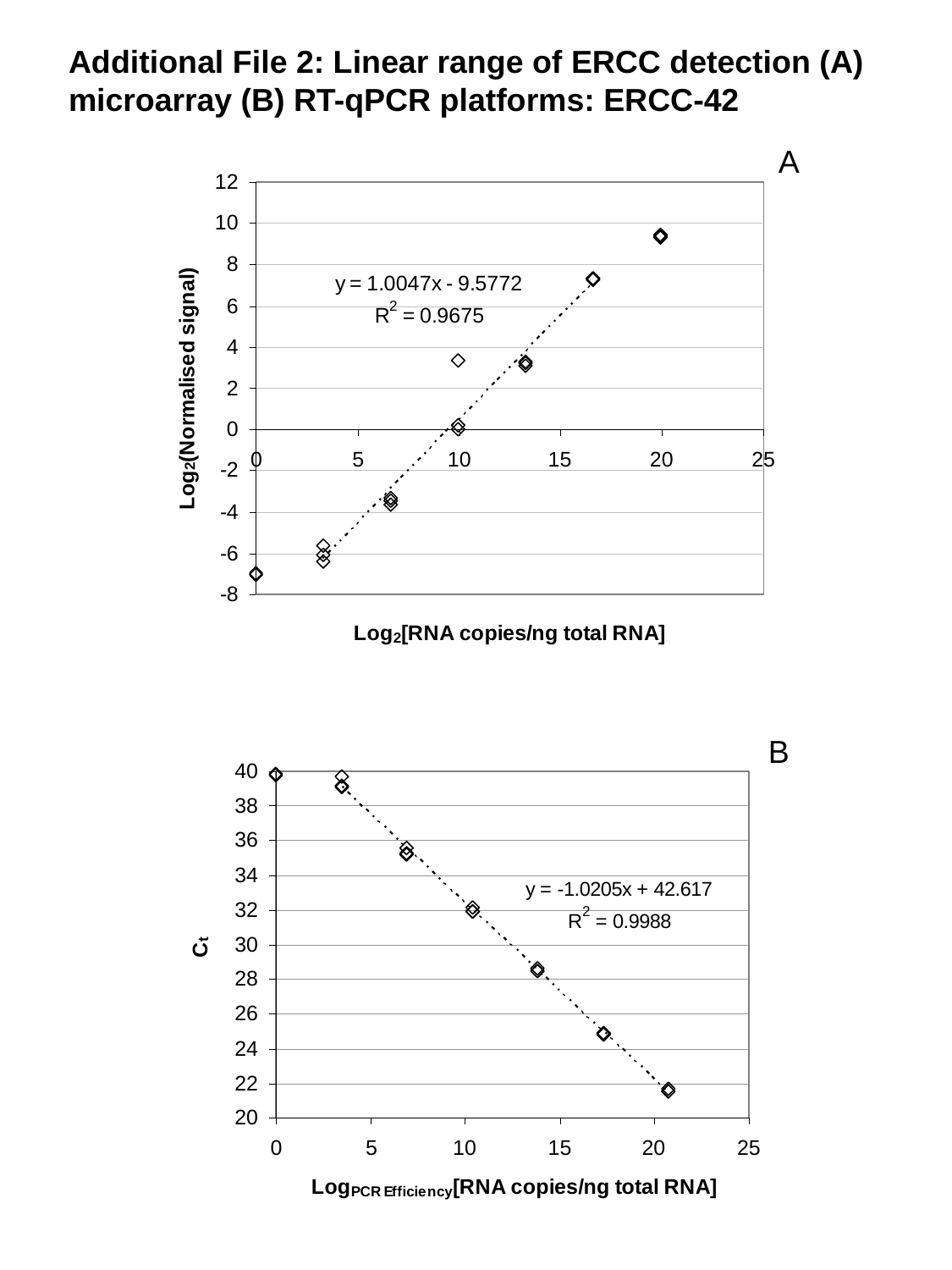

Additional File 2: Linear range of ERCC detection (A) microarray (B) RT-qPCR platforms: ERCC-42
A
B

## Slide 2
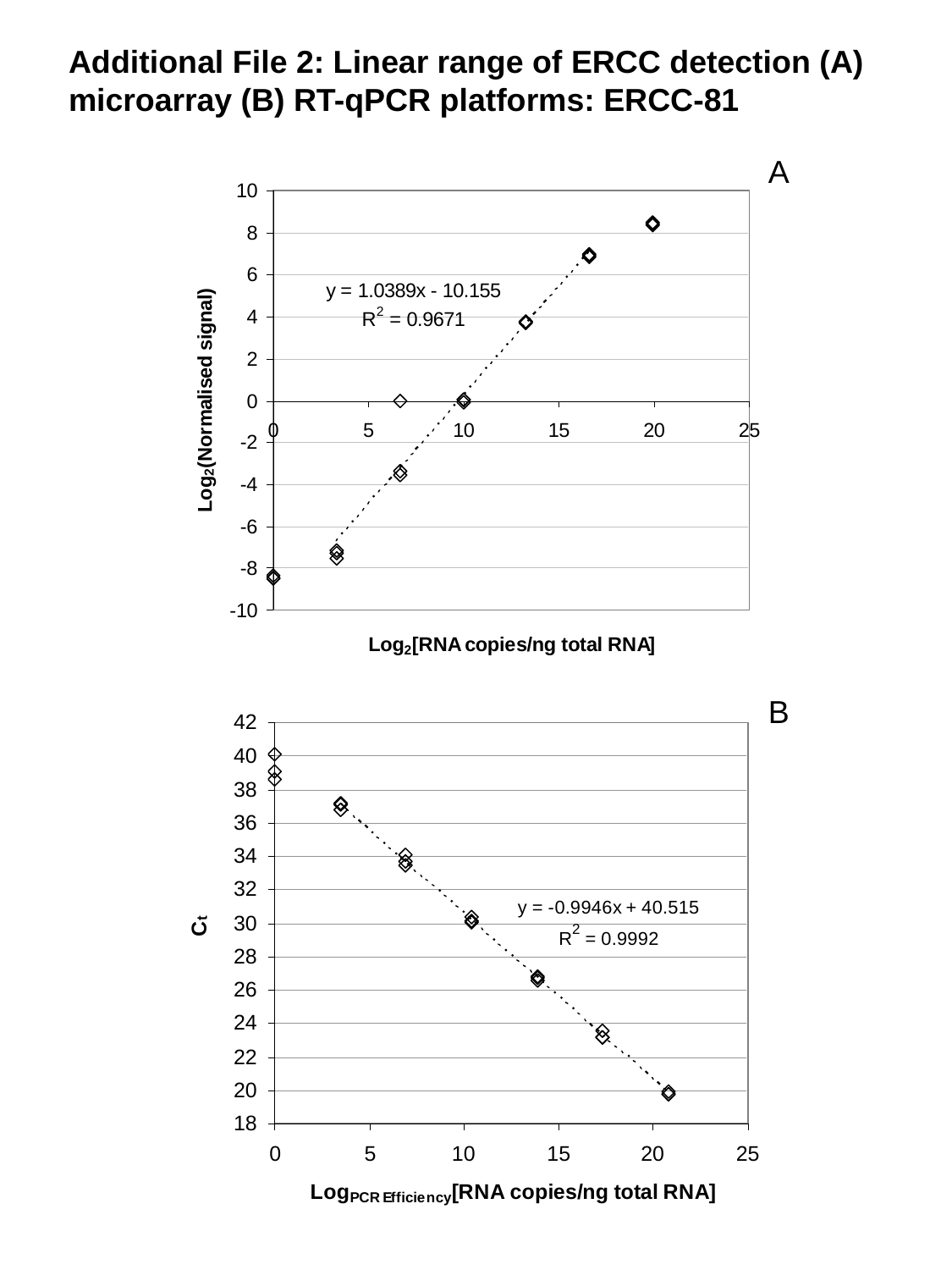

Additional File 2: Linear range of ERCC detection (A) microarray (B) RT-qPCR platforms: ERCC-81
A
B

## Slide 3
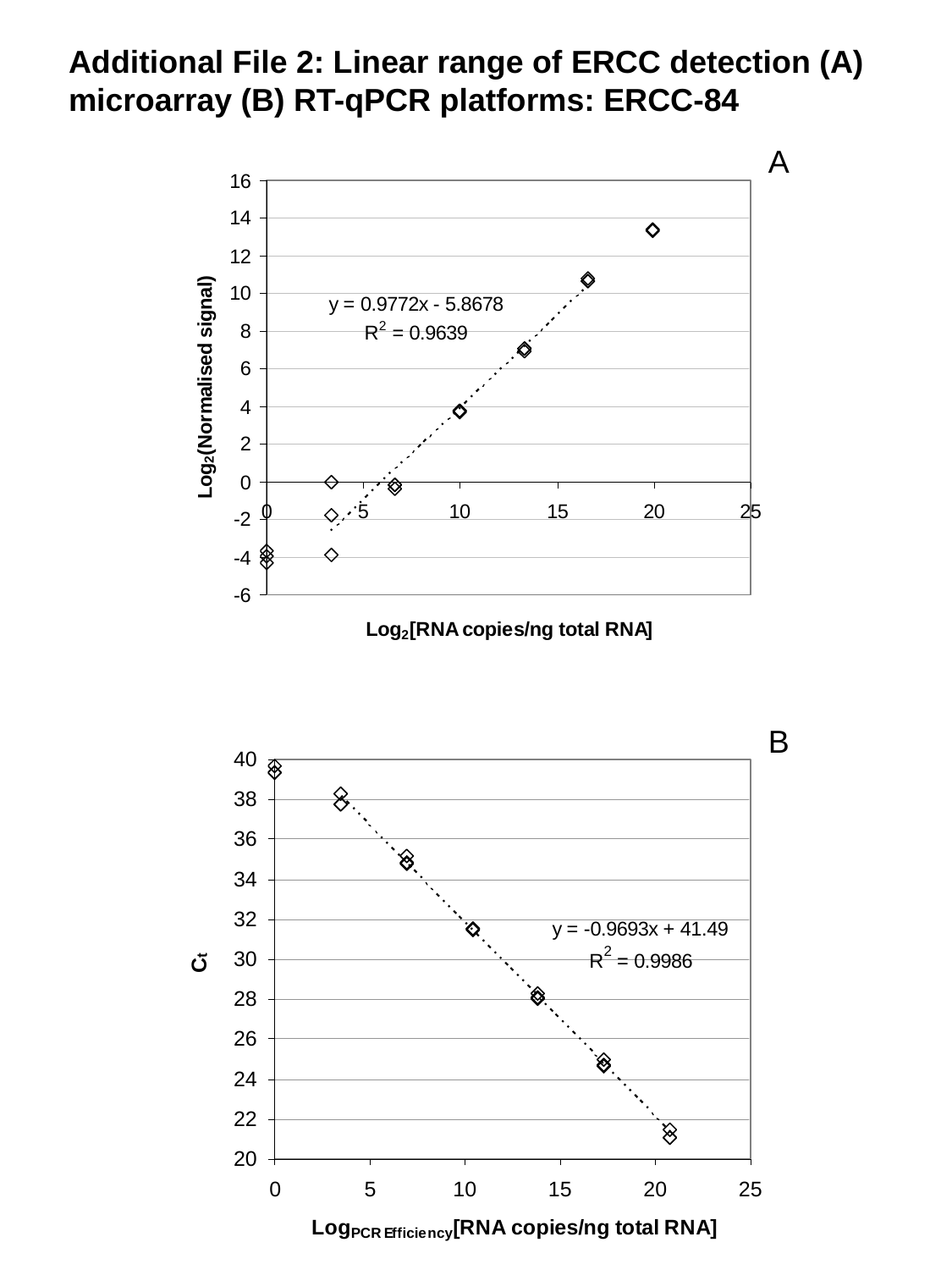

Additional File 2: Linear range of ERCC detection (A) microarray (B) RT-qPCR platforms: ERCC-84
A
B

## Slide 4
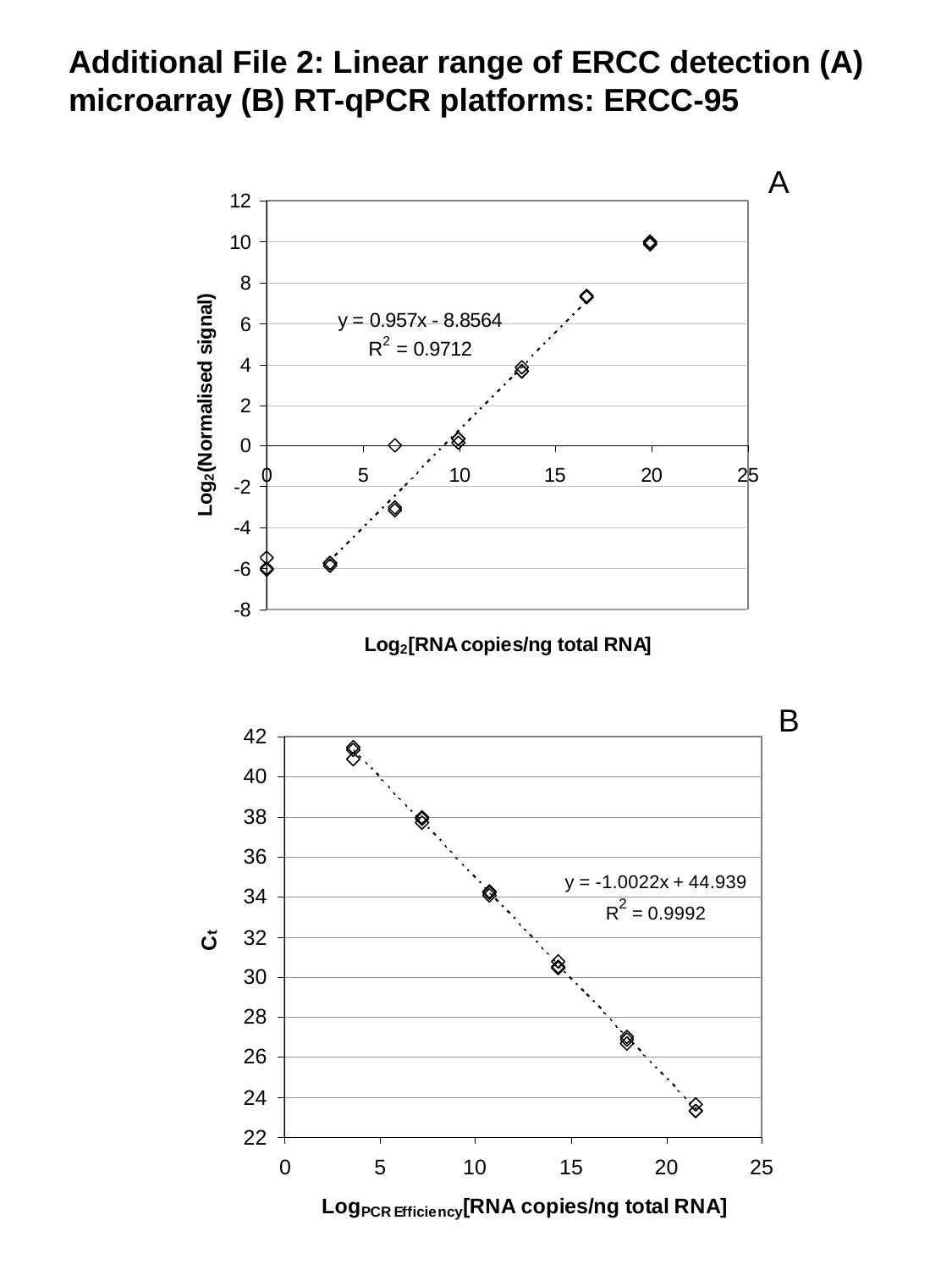

Additional File 2: Linear range of ERCC detection (A) microarray (B) RT-qPCR platforms: ERCC-95
A
B

## Slide 5
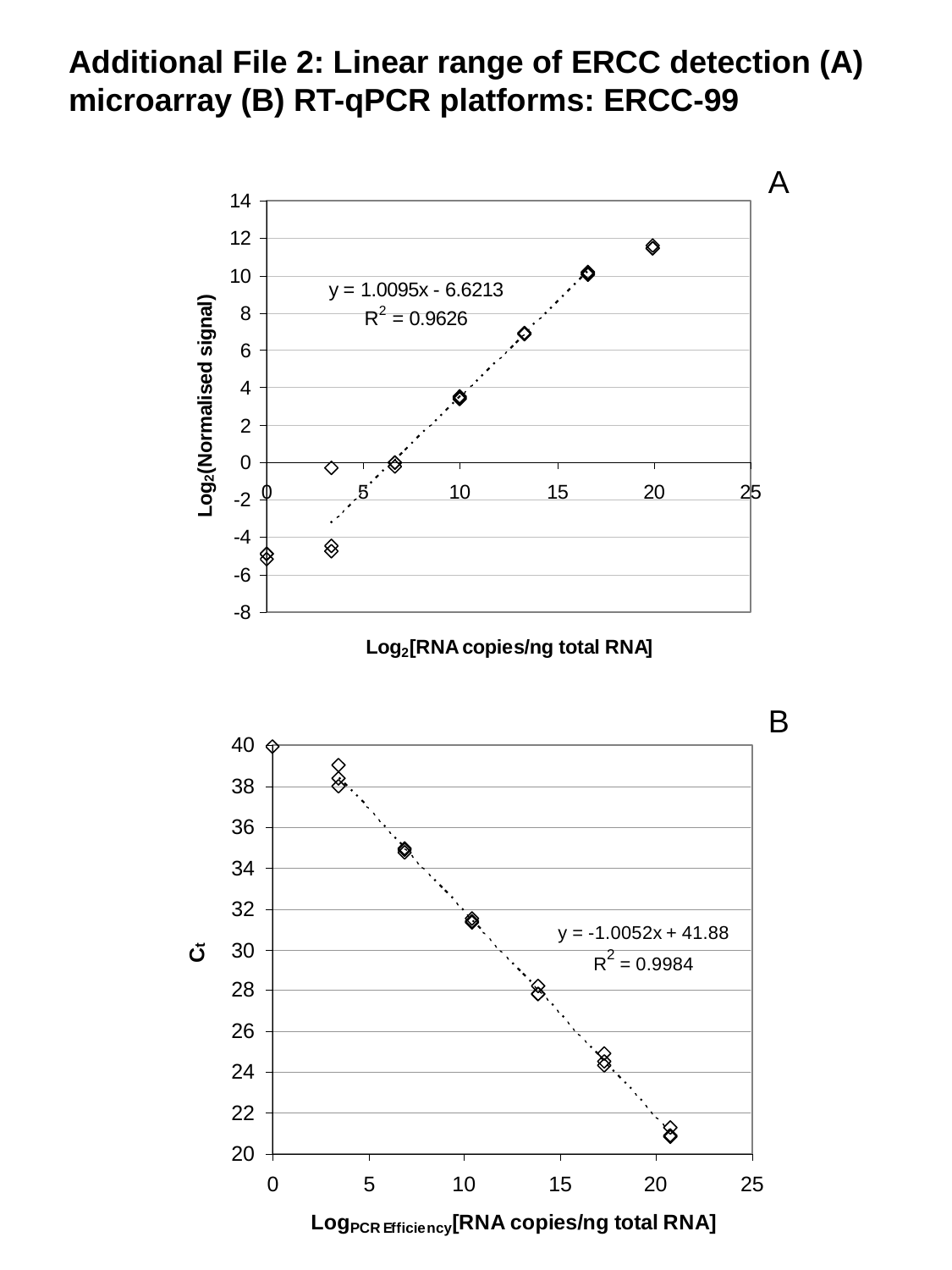

Additional File 2: Linear range of ERCC detection (A) microarray (B) RT-qPCR platforms: ERCC-99
A
B

## Slide 6
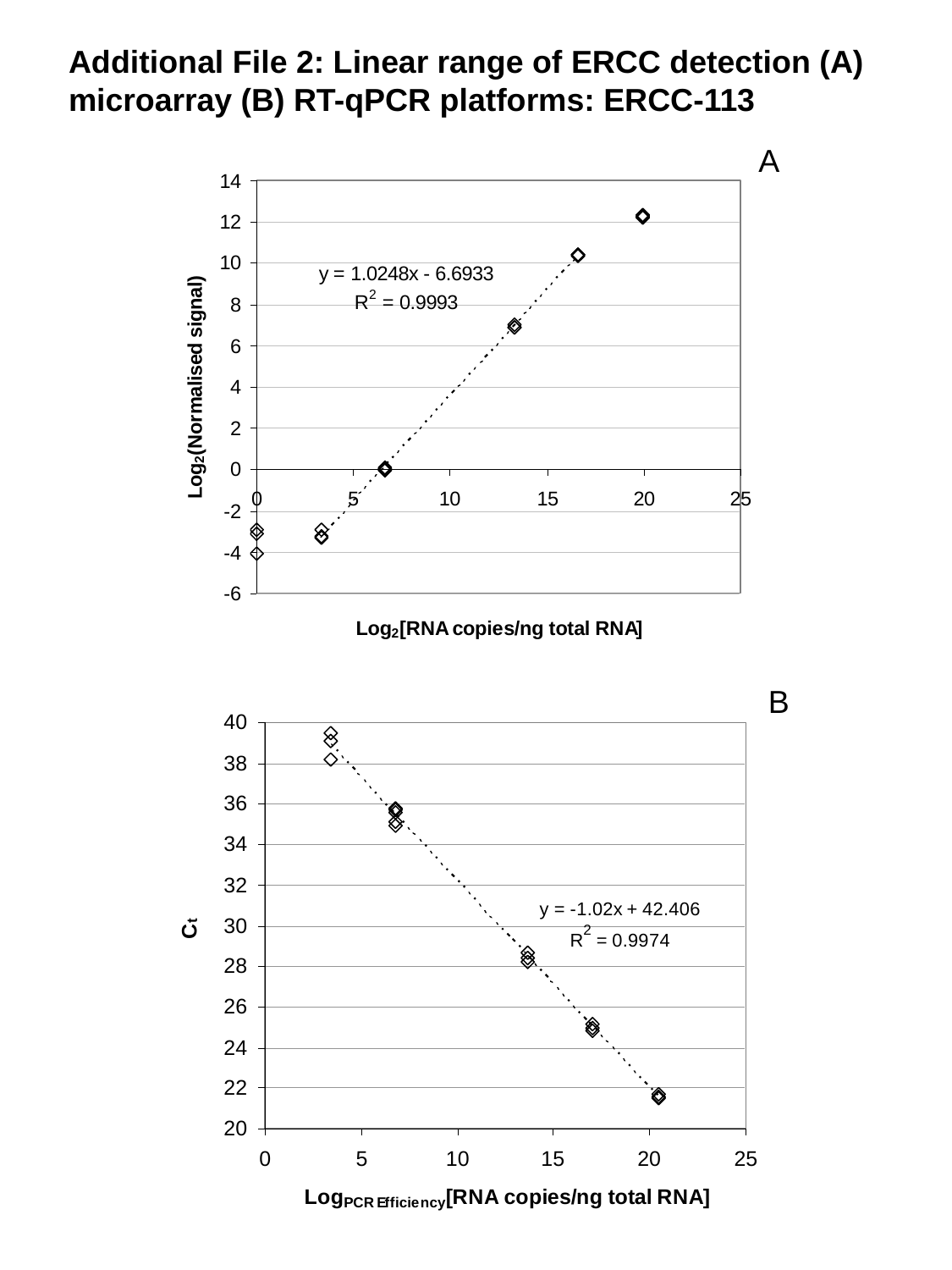

Additional File 2: Linear range of ERCC detection (A) microarray (B) RT-qPCR platforms: ERCC-113
A
B

## Slide 7
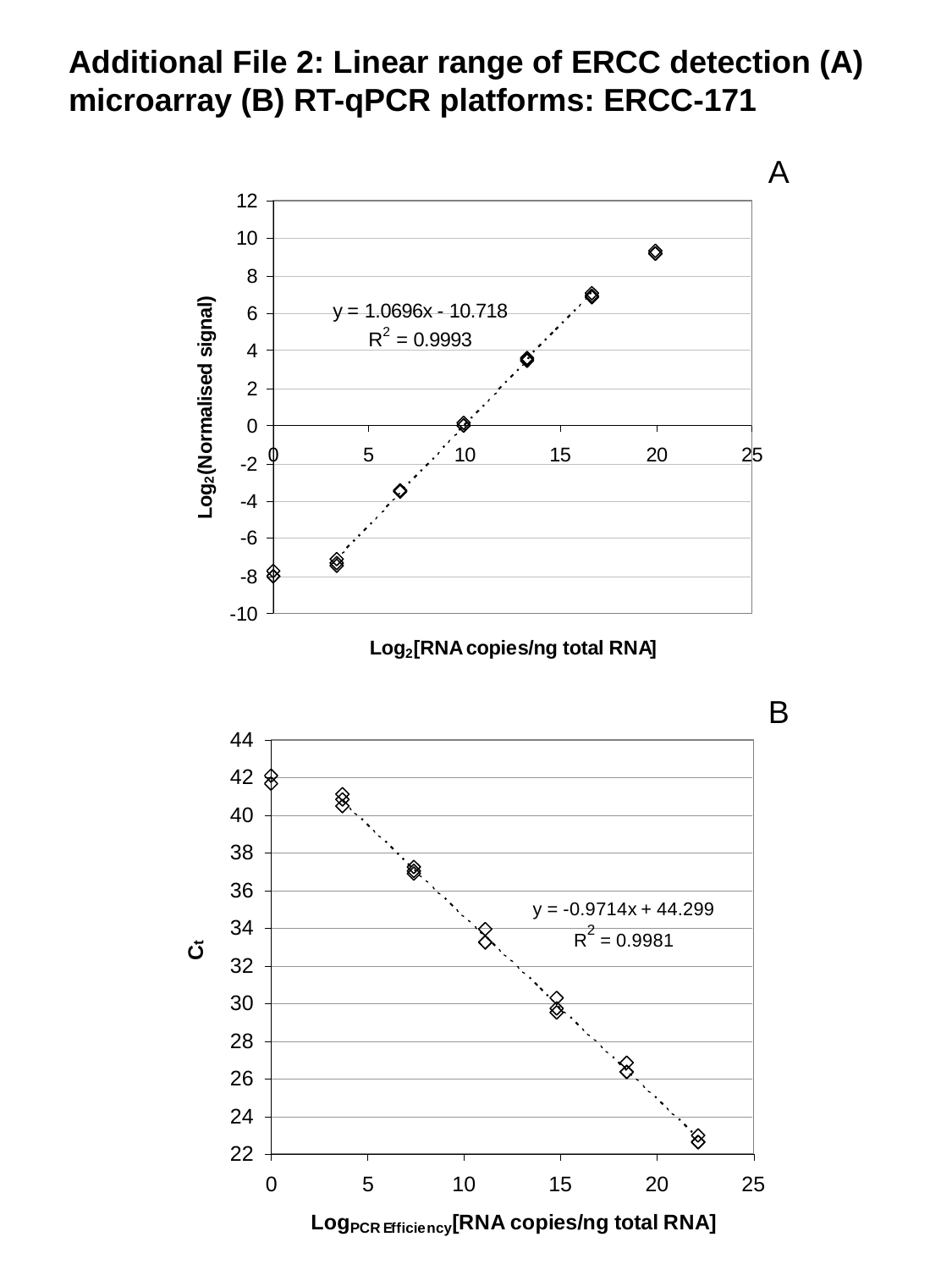

Additional File 2: Linear range of ERCC detection (A) microarray (B) RT-qPCR platforms: ERCC-171
A
B
