# Supplementary material for: Evaluation of external RNA controls for the standardisation of gene expression biomarker measurements
Source: BMC Genomics. 2010 Nov 24;11:662. doi: 10.1186/1471-2164-11-662 (PMC3091780; doi:10.1186/1471-2164-11-662)
Supplement: Additional file 3 — Results of correlation analysis of whole array data. Microsoft Word file displays data from pairwise correlation of microarray data (all entities) from three replicate microarrays. [file 1471-2164-11-662-S3.DOC]

### Additional file 3 – Results of correlation analysis of whole array data

### Pairwise correlation of microarray data (all entities) from 3 replicate microarrays

| **Sample No.** | **Run** | **1** | **2** | **3** |
| --- | --- | --- | --- | --- |
| **1** | **1** |  | 0.998 | 0.99716 |
| **1** | **2** | 0.998 |  | 0.998625 |
| **1** | **3** | 0.99716 | 0.998625 |  |
| **2** | **2** |  |  | 0.998565 |
| **2** | **3** |  | 0.998565 |  |
| **3** | **1** |  | 0.997777 | 0.985908 |
| **3** | **2** | 0.997777 |  | 0.986227 |
| **3** | **3** | 0.985908 | 0.986227 |  |
| **4** | **1** |  | 0.997306 | 0.997755 |
| **4** | **2** | 0.997306 |  | 0.996558 |
| **4** | **3** | 0.997755 | 0.996558 |  |
| **5** | **1** |  | 0.996369 | 0.997483 |
| **5** | **2** | 0.996369 |  | 0.997313 |
| **5** | **3** | 0.997483 | 0.997313 |  |
| **6** | **1** |  | 0.996078 | 0.996176 |
| **6** | **2** | 0.996078 |  | 0.998544 |
| **6** | **3** | 0.996176 | 0.998544 |  |
| **7** | **1** |  | 0.998435 | 0.994999 |
| **7** | **2** | 0.998435 |  | 0.996094 |
| **7** | **3** | 0.994999 | 0.996094 |  |
| **8** | **1** |  | 0.996752 | 0.996941 |
| **8** | **2** | 0.996752 |  | 0.996829 |
| **8** | **3** | 0.996941 | 0.996829 |  |

Pairwise-correlation analysis between replicate microarray measurements of the same sample was performed using all entities present on the Agilent 4 x 44K microarrays. Sample composition is as delineated in Table 1. Three independent hybridizations were performed. Pearson’s correlation coefficient (R^2^) values are displayed for each pair-wise comparison. Grey shading indicates missing data due to failed hybridization and black shading indicates the comparison is not applicable.
